# Risk-adjustment models for heart failure patients’ 30-day mortality and readmission rates: the incremental value of clinical data abstracted from medical charts beyond hospital discharge record

**DOI:** 10.1186/s12913-016-1731-9

**Published:** 2016-09-06

**Authors:** Jacopo Lenzi, Vera Maria Avaldi, Tina Hernandez-Boussard, Carlo Descovich, Ilaria Castaldini, Stefano Urbinati, Giuseppe Di Pasquale, Paola Rucci, Maria Pia Fantini

**Affiliations:** 1 Department of Biomedical and Neuromotor Sciences, Alma Mater Studiorum – University of Bologna, via San Giacomo 12, 40126 Bologna, Italy; 2 Department of Surgery, Stanford University, 300 Pasteur Drive, Stanford, CA 94305-2200 USA; 3 Department of Clinical Governance, Bologna Local Healthcare Authority, via Castiglione 29, 40124 Bologna, Italy; 4 Department of Programming and Control, Bologna Local Healthcare Authority, via Castiglione 29, 40124 Bologna, Italy; 5 Department of Cardiology, Bellaria Hospital, via Altura 3, 40139 Bologna, Italy; 6 Department of Cardiology, Maggiore Hospital, Largo Nigrisoli 2, 40133 Bologna, Italy

**Keywords:** Heart failure, Risk-adjustment, Mortality, Readmissions

## Abstract

**Background:**

Hospital discharge records (HDRs) are routinely used to assess outcomes of care and to compare hospital performance for heart failure. The advantages of using clinical data from medical charts to improve risk-adjustment models remain controversial. The aim of the present study was to evaluate the additional contribution of clinical variables to HDR-based 30-day mortality and readmission models in patients with heart failure.

**Methods:**

This retrospective observational study included all patients residing in the Local Healthcare Authority of Bologna (about 1 million inhabitants) who were discharged in 2012 from one of three hospitals in the area with a diagnosis of heart failure. For each study outcome, we compared the discrimination of the two risk-adjustment models (i.e., HDR-only model and HDR-clinical model) through the area under the ROC curve (AUC).

**Results:**

A total of 1145 and 1025 patients were included in the mortality and readmission analyses, respectively. Adding clinical data significantly improved the discrimination of the mortality model (AUC = 0.84 vs. 0.73, *p* < 0.001), but not the discrimination of the readmission model (AUC = 0.65 vs. 0.63, *p* = 0.08).

**Conclusions:**

We identified clinical variables that significantly improved the discrimination of the HDR-only model for 30-day mortality following heart failure. By contrast, clinical variables made little contribution to the discrimination of the HDR-only model for 30-day readmission.

**Electronic supplementary material:**

The online version of this article (doi:10.1186/s12913-016-1731-9) contains supplementary material, which is available to authorized users.

## Background

Heart failure (HF) is a complex syndrome characterized by high mortality and morbidity and is a leading cause of hospitalization [1]. Aging population, decreased HF mortality due to improvement of therapeutic interventions, effective secondary prevention, and hospital and primary care management strategies have led to an increased burden of HF on healthcare systems [1, 2].

Assessing quality of care, especially outcomes of care, and comparing hospital performance have become important issues needed to ensure a healthcare system that is cost-effective for HF [3, 4]. To this end, statistical models to compare hospital performance across important outcomes must adjust for differences in demographic and clinical characteristics, as the case mix of patients may vary among regions and hospitals [5, 6].

Hospital discharge records (HDRs) (also known as administrative claims databases) are a main source of data for outcomes studies because data collection is inexpensive and they enable the analysis of large populations and a large number of conditions and pathologies [7]; however, these data lack clinical granularity and do not allow one to determine the severity and history of disease [5–8]. In Italy, the National Outcome Evaluation Program (*Programma Nazionale Esiti* – PNE), an initiative endorsed by the National Agency for Regional Health Services (*Agenzia Nazionale per i Servizi Sanitari Regionali*–AGENAS) that monitors healthcare outcomes across hospitals and municipalities, routinely uses HDRs to derive hospital-specific indicators and important patient characteristics used in risk-adjustment of different patient populations [9].

Many authors have underlined the advantages of adding specific clinical data to HDR-based risk-adjustment models [8, 10–13]: the integration of clinical data with HDRs in risk-adjustment models could improve the predictive power and the control of confounding, and identify variables that mainly influence the outcome [13]. Medical charts could indeed offer important information on the patient’s clinical conditions unavailable in HDRs that allow one to differentiate between comorbidities and complications [8].

With regard to HF, a recent study [14] suggested that the addition of clinical data to HDR-based models improved the discrimination of mortality risk-adjustment models and shifted mortality performance rankings in inter-hospital comparison. However, clinical data did not substantially improve the discrimination of the readmission risk model nor the hospital ranking. Another study [5] found no difference between HDR- and clinical-based predictive models for 30-day mortality. Overall, a recent systematic review of the literature revealed that the discriminatory ability of the models was generally higher for the prediction of death than for the prediction of hospital readmission [15].

The impact of clinical data in risk-adjustment models is therefore controversial because, even though clinical data add information, they do not always improve the discrimination of the models. Moreover, the availability and reliability of clinical data may vary greatly among hospitals, and their collection entails more effort and costs than HDRs data. For this reason, it is useful to identify a limited number of clinical variables that are significantly correlated with HF outcomes and are affordable and easy to collect [7]. The aim of this study was to evaluate the usefulness of clinical variables and drug prescriptions in predicting 30-day mortality and 30-day readmissions in patients with HF.

## Methods

### Setting and study population

This retrospective observational study included all patients residing in the Local Healthcare Authority (LHA) of Bologna who were discharged from one of three public hospitals in the area (hereinafter called “A”, “B” and “C”) between December 2, 2011 and December 1, 2012 with a primary diagnosis of HF (ICD-9-CM diagnosis codes: 398.91, 402.x1, 404.x1, 404.x3, 428.xx). Data were retrieved from the HDRs Database (see Additional file 1 for a description of the data source).

Hospital A is the second largest hospital of Bologna, with more than 900 beds and about 40 wards; hospital B, also located in Bologna, has about 370 bed and 20 wards, and is a center of excellence in the field of neuroscience; hospital C is located in a municipality not far from Bologna, with about 200 beds and 15 wards, and is the referral facility in the northern LHA area.

Patients were excluded if any of the following criteria were met:

1. A secondary diagnosis of non-cardiogenic acute pulmonary edema or acute kidney failure (ICD-9-CM diagnosis codes: 518.4, 584.x), i.e., patients with symptoms probably related to causes other than HF, in keeping with the PNE definition to allow comparison of results;
2. Age <18 or >100 years, because very young and very old patients may have distinctive clinical features at diagnosis and survival;
3. Transfer from another facility, to ascribe the study outcomes to the hospital where the patient was initially admitted;
4. Incomplete clinical data (i.e., missing laboratory data, electrocardiography, etc.), to ensure comparability of risk-adjustment models. We decided to exclude these patients because the pattern of missing clinical data appeared to be independent of patient’s age, gender and length of stay (data not shown).

For patients with multiple eligible hospital admissions over the 1-year study period, we considered only the first one as the index admission because hospitalizations of the same patient are correlated, thus violating the assumption of independence required by regression models. Still, we are aware that excluding multiple readmissions, i.e., hospital admissions of patients with presumably chronic HF, may limit the generalizability of the results.

### Data

Variables considered for inclusion in risk-adjustment models were retrieved from three data sources: (1) HDRs, (2) Outpatient Pharmaceutical Database (OPD), and (3) medical charts (see Additional file 1 for a description of the data sources).

Variables retrieved from the HDRs Database were: age, gender, length of stay, and 23 comorbidities chosen a priori and identified in the index hospitalization and in all hospital admissions occurring two-years prior to the index hospitalization (see Additional file 2, which includes the detailed list of comorbidities). Moreover, we collected from the OPD information on filled prescriptions of: antidiabetic drugs (ATC code A10), drugs for cardiac therapy (C01), drugs for obstructive airway diseases (R03), diuretics (C03), β-blockers (C07), angiotensin-converting enzyme inhibitors/angiotensin receptor blockers (C09), calcium channel blockers and/or other antihypertensive drugs (C08, C02), statins (C10AA), and antiplatelet drugs (B01AC). Treatment for each drug was defined as at least one filled prescription in the three months preceding the HF hospital admission.

Clinical data abstracted from medical chart review were:

1. Emergency department utilization (yes/no);
2. Heart rate (bpm) and systolic blood pressure (mmHg) at hospital admission;
3. Pulmonary congestion (yes/no), determined with radiography at hospital admission;
4. Heart rhythm (sinus rhythm/atrial fibrillation/pacemaker rhythm), bundle branch block (no/right/left), and QRS complex (only for patients with left bundle branch block) retrieved from electrocardiography at hospital admission;
5. Serum creatinine (mg/dL), sodium (mmol/L), and hemoglobin (g/dL) at hospital admission;
6. Previously implanted cardiac devices, including cardiac resynchronization therapies (yes/no): pacemaker, implantable cardioverter defibrillator (ICD).

We selected these clinical data for three main reasons. First, other studies have shown that these data are predictive of mortality and/or readmission among patients with HF [15–21]. Second, most of these measures are quantitative measures that are not captured in HDRs. Lastly these data reflect multiple clinical domains, including laboratory test results, diagnostic test results and vital signs.

The data collection for the present study consisted of a thorough review of more than 1000 medical charts which, in Emilia-Romagna, are still paper-based. This review process was carried out by four medical residents in Public Health who had been previously trained by a team of cardiologists. The objective of this training was to test and improve the review of medical charts, and to ensure that medical residents would collect only clinical data determined at hospital admission, i.e., prior to any medical intervention. Medical residents worked always in pairs and imputed data abstracted from medical charts in a spreadsheet which was later linked with HDRs and OPD using the patient’s identification code.

### Study outcomes

The study had two main outcomes of interest. The first outcome was all-cause death within thirty days of index admission, identified through the Regional Mortality Register Database (see Additional file 1 for a description of the data source). The second outcome of interest was all-cause unplanned readmission to any Italian hospital between two to thirty days after the index discharge and lasting more than one day. For the readmission analysis we excluded patients deceased during index hospitalization, as they cannot experience rehospitalizations.

### Statistical analysis

Analysis of variance, Kruskal-Wallis test, *χ*^*2*^ test and Fisher’s exact test were used, where appropriate, to compare the distribution of patients’ demographic and clinical characteristics across hospitals.

#### Risk-adjustment models based on HDR variables

The crude association between each potential predictor and the study outcomes (30-day mortality and unplanned readmissions) was first examined in univariable logistic regression models. In these models, age and length of stay were transformed into normally distributed variables (cubed and log-transformed, respectively) in order to linearize their relationship with the logistic link function [22]. Predictors significantly associated with the outcome at *p* <0.25 in univariable analyses were selected for inclusion in multivariable logistic regression models. A bootstrap procedure was used to determine which of these factors were significantly associated with the outcome in multivariable models. Using this approach, 1000 replicated bootstrap samples were selected from the original cohort. In each replicated sample, age and gender were forced into the model, and a backward elimination of potential confounders was applied with a significance level of removal equal to 0.05. Only risk factors selected in at least 50 % of the replicates were included as covariates in the final multivariable logistic regression models.

#### Risk-adjustment models based on HDR plus OPD plus medical charts variables

The steps described above to build the HDR-only model were replicated by adding the variables retrieved from OPD and medical charts to HRD variables. Systolic blood pressure was log-transformed and serum creatinine was transformed using the reciprocal of square root to normalize their distribution.

For each study outcome, we compared the discrimination of the two risk-adjustment models through the area under the ROC curve (AUC). Discrimination is the ability of the model to distinguish between high-risk and low-risk patients. The incremental contribution of the variables retrieved from OPD plus medical charts was assessed also using the likelihood ratio (LR) test, because it has been demonstrated that the AUC test produces conservative test size and low power [23]. Lastly, the goodness of fit of each model was estimated using the coefficient of determination (McFadden’s adjusted pseudo *R*^*2*^) and the Akaike Information Criterion (AIC).

Statistical analyses were carried out using the Stata software package, version 13 (StataCorp. 2013. *Stata Statistical Software: Release 13*. College Station, TX: StataCorp LP).

## Results

**Fig. 1:**
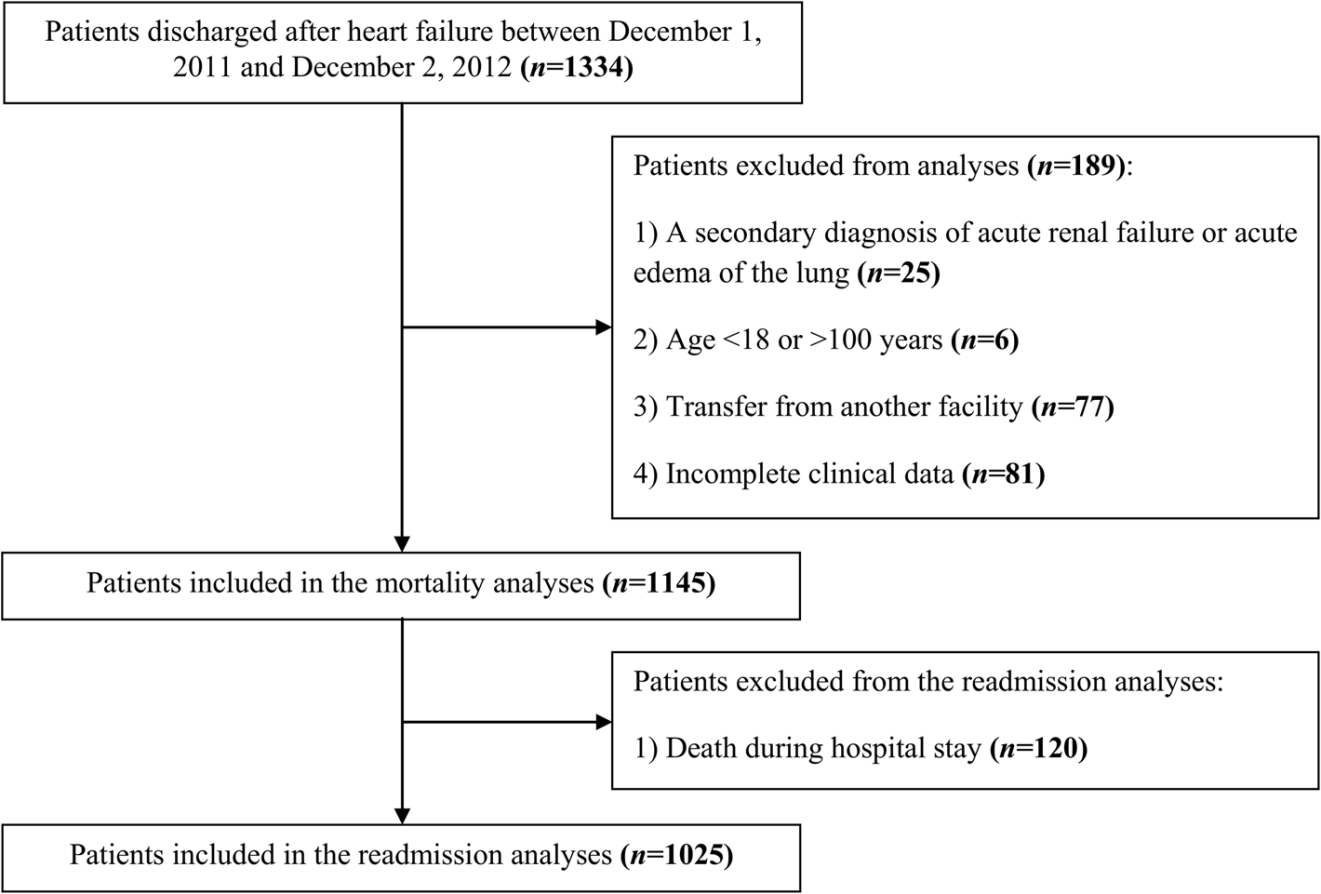
Patients’ flow diagram

Of the 1334 patients discharged after HF, 1145 (85.8 %) met inclusion criteria for the mortality analyses, while 1025 (76.8 %) met inclusion criteria for the readmission analyses (Fig. 1). The overall 30-day mortality and 30-day readmission rates were 13.2 % (151 patients out of 1145) and 15.6 % (160 patients out of 1025), respectively. Of the 151 deaths within 30 days of hospital admission, 15 (9.9 %) occurred on the day of admission, and 37 (24.5 %) occurred after hospital discharge. Of the 160 patients rehospitalized within 30 days of discharge, 55 (34.4 %) were readmitted to the hospital for another episode of HF, and 21 (13.1 %) for cardiovascular causes other than HF.

The crude 30-day mortality rates in hospitals A, B and C were 16.0, 2.1 and 8.7 %, while the crude 30-day readmission rates were 15.2, 24.2 and 13.5 %, respectively.

The distribution of patient characteristics, overall and by hospital, is reported in Table 1. Mean age was 81 years, 54.3 % were female, and median length of stay was 7 days. The most frequent comorbid conditions were hypertension (32.7 %), other forms of ischemic heart disease (32.4 %), previous HF diagnosis (28.9 %), conduction disorders and cardiac dysrhythmias (27.7 %), and chronic nephropathies (26.6 %). Mean heart rate and systolic blood pressure were 87 ± 20.2 bpm and 140 ± 32.6 mmHg, respectively. Almost two thirds of patients (60.7 %) had used calcium channel blockers and/or other antihypertensive drugs before admission. Some differences in the case mix of patients discharged from the three hospitals were found; specifically, patients from hospital A were older and more frequently female, and had more often chronic nephropathies and less often pulmonary congestion; on the other hand, patients discharged from hospital B had more cardiovascular comorbidities and previous device implantation, and used less frequently emergency department.

**Table 1:** Distribution of patient characteristics collected from HDRs, OPD and medical charts, overall and by hospital

Patient characteristics	Hospital A		Hospital B		Hospital C		All		*p*
	(*n* = 786)		(*n* = 96)		(*n* = 263)		(*n* = 1145)		
From HDRs									
Age in years, mean [SD]	82.3	[9.2]	77.1	[11.0]	79.7	[10.2]	81.3	[9.8]	<0.001
Gender, n (%)									0.01
Male	339	(43.1)	55	(57.3)	129	(49.0)	523	(45.7)	
Female	447	(56.9)	41	(42.7)	134	(51.0)	622	(54.3)	
Length of stay in days, median [IQR]	8	[7]	9.5	[9.5]	8	[6]	8	[7]	<0.001
Comorbidity, n (%)									
Malignant tumors	75	(9.5)	11	(11.5)	31	(11.8)	117	(10.2)	0.53
Diabetes	129	(16.4)	19	(19.8)	49	(18.6)	197	(17.2)	0.56
Disorders of lipoid metabolism	44	(5.6)	6	(6.3)	27	(10.3)	77	(6.7)	0.03
Obesity	23	(2.9)	2	(2.1)	13	(4.9)	38	(3.3)	0.26
Hematologic diseases	127	(16.2)	10	(10.4)	50	(19.0)	187	(16.3)	0.14
Hypertensive diseases	247	(31.4)	31	(32.3)	96	(36.5)	374	(32.7)	0.31
Previous AMI	122	(15.5)	25	(26.0)	48	(18.3)	195	(17.0)	0.03
Other forms of ischemic heart disease	257	(32.7)	42	(43.8)	72	(27.4)	371	(32.4)	0.01
Ill-defined descriptions and complications of heart disease	12	(1.5)	4	(4.2)	6	(2.3)	22	(1.9)	0.14
Rheumatic heart disease	50	(6.4)	9	(9.4)	15	(5.7)	74	(6.5)	0.45
Cardiomyopathies	56	(7.1)	21	(21.9)	19	(7.2)	96	(8.4)	<0.001
Other cardiac diseases	60	(7.6)	21	(21.9)	34	(12.9)	115	(10.0)	<0.001
Conduction disorders and cardiac dysrhythmias	207	(26.3)	30	(31.3)	80	(30.4)	317	(27.7)	0.32
Cerebrovascular diseases	121	(15.4)	21	(21.9)	50	(19.0)	192	(16.8)	0.15
Vascular diseases	66	(8.4)	11	(11.5)	26	(9.9)	103	(9.0)	0.52
COPD	122	(15.5)	19	(19.8)	42	(16.0)	183	(16.0)	0.56
Chronic nephropathies	173	(22.0)	34	(35.4)	97	(36.9)	304	(26.6)	<0.001
Chronic diseases of liver, pancreas and intestine	16	(2.0)	1	(1.0)	5	(1.9)	22	(1.9)	>0.99
Previous bypass	14	(1.8)	6	(6.3)	18	(6.8)	38	(3.3)	<0.001
Previous PCI	40	(5.1)	9	(9.4)	16	(6.1)	65	(5.7)	0.20
Other surgery of the heart	18	(2.3)	9	(9.4)	7	(2.7)	34	(3.0)	<0.01
Other surgery of great vessels	18	(2.3)	2	(2.1)	8	(3.0)	28	(2.4)	0.74
Previous diagnosis of heart failure	223	(28.4)	34	(35.4)	74	(28.1)	331	(28.9)	0.34
From OPD									
Previous medication use, n (%)									
Antidiabetic drugs	174	(22.1)	24	(25.0)	59	(22.4)	257	(22.4)	0.79
Drugs for cardiac therapy	262	(33.3)	25	(26.0)	70	(26.6)	357	(31.2)	0.07
Drugs for obstructive airway diseases	163	(20.7)	30	(31.3)	70	(26.6)	263	(23.0)	0.02
Diuretics	457	(58.1)	59	(61.5)	159	(60.5)	675	(59.0)	0.70
β-blockers	200	(25.4)	32	(33.3)	75	(28.5)	307	(26.8)	0.20
ACEIs/ARBs	366	(46.6)	38	(39.6)	115	(43.7)	519	(45.3)	0.36
Calcium channel blockers and/or other antihypertensive drugs	472	(60.1)	68	(70.8)	155	(58.9)	695	(60.7)	0.10
Statins	383	(48.7)	53	(55.2)	131	(49.8)	567	(49.5)	0.49
Antiplatelet drugs	181	(23.0)	25	(26.0)	56	(21.3)	262	(22.9)	0.63
From medical charts									
Emergency department utilization, n (%)	767	(97.6)	78	(81.3)	256	(97.3)	1101	(96.2)	<0.001
Heart rate in bpm, mean [SD]^a^	87	[20.7]	86	[19.4]	85	[18.7]	87	[20.2]	0.52
Systolic blood pressure in mmHg, mean [SD]	140	[32.8]	139	[32.5]	142	[32.3]	140	[32.6]	0.67
Pulmonary congestion, n (%)	666	(84.7)	87	(90.6)	241	(91.6)	994	(86.8)	<0.001
Heart rhythm, n (%)									0.14
Sinus rhythm	405	(51.5)	42	(43.8)	127	(48.3)	574	(50.1)	
Atrial fibrillation	323	(41.1)	41	(42.7)	118	(44.9)	482	(42.1)	
Pacemaker rhythm	55	(7.0)	13	(13.5)	18	(6.8)	86	(7.5)	
Bundle branch block, n (%)									0.55
No	625	(79.5)	78	(81.3)	208	(79.1)	911	(79.6)	
Right	72	(9.2)	6	(6.3)	30	(11.4)	108	(9.4)	
Left	89	(11.3)	12	(12.5)	25	(9.5)	126	(11.0)	
QRS complex, mean [SD]^b^	151	[18.1]	159	[24.0]	152	[19.9]	152	[19.0]	0.25
Serum creatinine in mg/dL, mean [SD]	1.42	[0.9]	1.32	[0.6]	1.4	[0.9]	1.41	[0.9]	0.59
Chronic kidney disease stage (using MDRD formula), n (%)									0.38
1	60	(7.6)	9	(9.4)	32	(12.2)	101	(8.8)	
2	210	(26.7)	25	(26.0)	74	(28.1)	309	(27.0)	
3a	191	(24.3)	24	(25.0)	53	(20.2)	268	(23.4)	
3b	179	(22.8)	27	(28.1)	60	(22.8)	266	(23.2)	
4	122	(15.5)	10	(10.4)	35	(13.3)	167	(14.6)	
5	24	(3.1)	1	(1.0)	9	(3.4)	34	(3.0)	
Sodium in mmol/l, mean [SD]	139	[5.8]	139	[3.7]	138	[4.8]	139	[5.4]	<0.001
Hemoglobin in g/dL, mean [SD]	12.1	[2.2]	12.1	[1.9]	12.1	[2.0]	12.1	[2.1]	>0.90
Previously implanted pacemaker, n (%)	80	(10.2)	21	(21.9)	29	(11.0)	130	(11.4)	<0.01
Previously implanted cardioverter defibrillator, n (%)	13	(1.7)	10	(10.4)	9	(3.4)	32	(2.8)	<0.001

*Abbreviations*: *HDR* Hospital Discharge Record, *SD* standard deviation, *IQR* interquartile range, *AMI* acute myocardial infarction, *COPD* chronic obstructive pulmonary disease, *PCI* percutaneous coronary intervention, *OPD* Outpatient Pharmaceutical Database, *ACEI/ARB* angiotensin-converting enzyme inhibitor/angiotensin receptor blocker, *MDRD* Modification of Diet in Renal Disease

^a^Only for patients with sinus rhythm

^b^Only for patients with left bundle branch block

### Comparison of risk-adjustment models

**Fig. 2:**
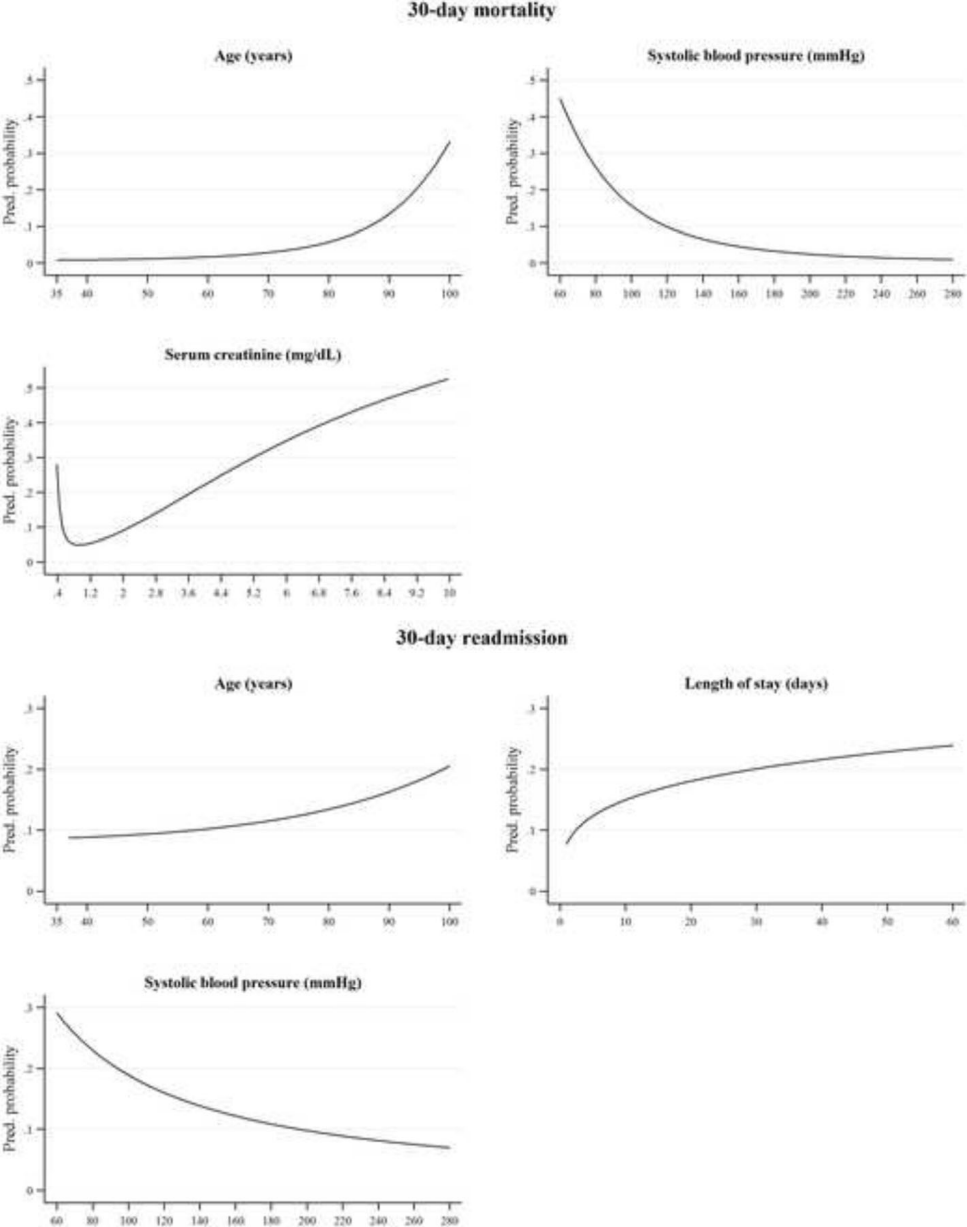
Predicted probabilities of 30-day mortality and readmission for each of the continuous variables retained in the HDR-plus-clinical multivariable risk-adjustment models

Table 2 reports the variables retained in the risk-adjustment models based on HDRs only (models #1) and HDR plus clinical data (models #2). Figure 2 illustrates, for each of the continuous variables retained in models #2, the predicted probabilities of death and readmission.

**Table 2:** Odds ratios (ORs) of 30-day mortality and readmission for each of the variables retained in multivariable risk-adjustment models based on HDRs only (#1) and HDR plus clinical data (#2)

Characteristics	Model #1			Model #2		
	OR	95 % CI	*P*	OR	95 % CI	*p*
30-day mortality						
From HDRs						
Gender						
Male	1.00			1.00		
Female	0.58	0.41–0.83	<0.01	0.63	0.37–1.06	0.08
Age^a^	1.00	1.00–1.00	<0.001	1.00	1.00–1.00	<0.001
Cerebrovascular disease						
No	1.00			1.00		
Yes	1.89	1.53–2.35	<0.001	1.96	1.33–2.87	<0.01
From medical charts						
Systolic blood pressure^b^	–			0.05	0.02–0.13	<0.001
Heart rhythm						
Sinus rhythm with heart rate < 90 bpm^c^	–			1.00		
Sinus rhythm with heart rate ≥ 90 bpm^c^	–			2.94	1.87–4.61	<0.001
Atrial fibrillation	–			1.78	1.31–2.41	<0.001
Pacemaker rhythm	–			1.21	0.29–5.11	0.79
Serum creatinine^d^	–			0.26	0.12–0.54	<0.001
30-day readmission						
From HDRs						
Gender						
Male	1.00			1.00		
Female	0.95	0.66–1.36	0.78	0.96	0.67–1.38	0.82
Age^a^	1.00	1.00–1.00	<0.01	1.00	1.00–1.00	<0.01
Length of stay^b^	1.41	1.14–1.74	<0.01	1.38	1.12–1.71	<0.01
Previous acute myocardial infarction						
No	1.00			1.00		
Yes	2.05	1.11–3.78	0.02	1.90	1.05–3.44	0.03
From medical charts						
Systolic blood pressure^b^	–			0.33	0.19–0.59	<0.001

*Abbreviations*: *CI* confidence interval

^a^This variable was cubed to achieve normality

^b^This variable was log-transformed to achieve normality

^c^This cutoff was chosen using ROC analysis to minimize the number of false positive and false negatives

^d^This variable was transformed using the reciprocal of square root to achieve normality. We also included a squared term in the regression to achieve a good model specification (OR = 357.41, 95 % CI = 36.36–3513.66, *p* < 0.001)

#### Thirty-day mortality

**Fig. 3:**
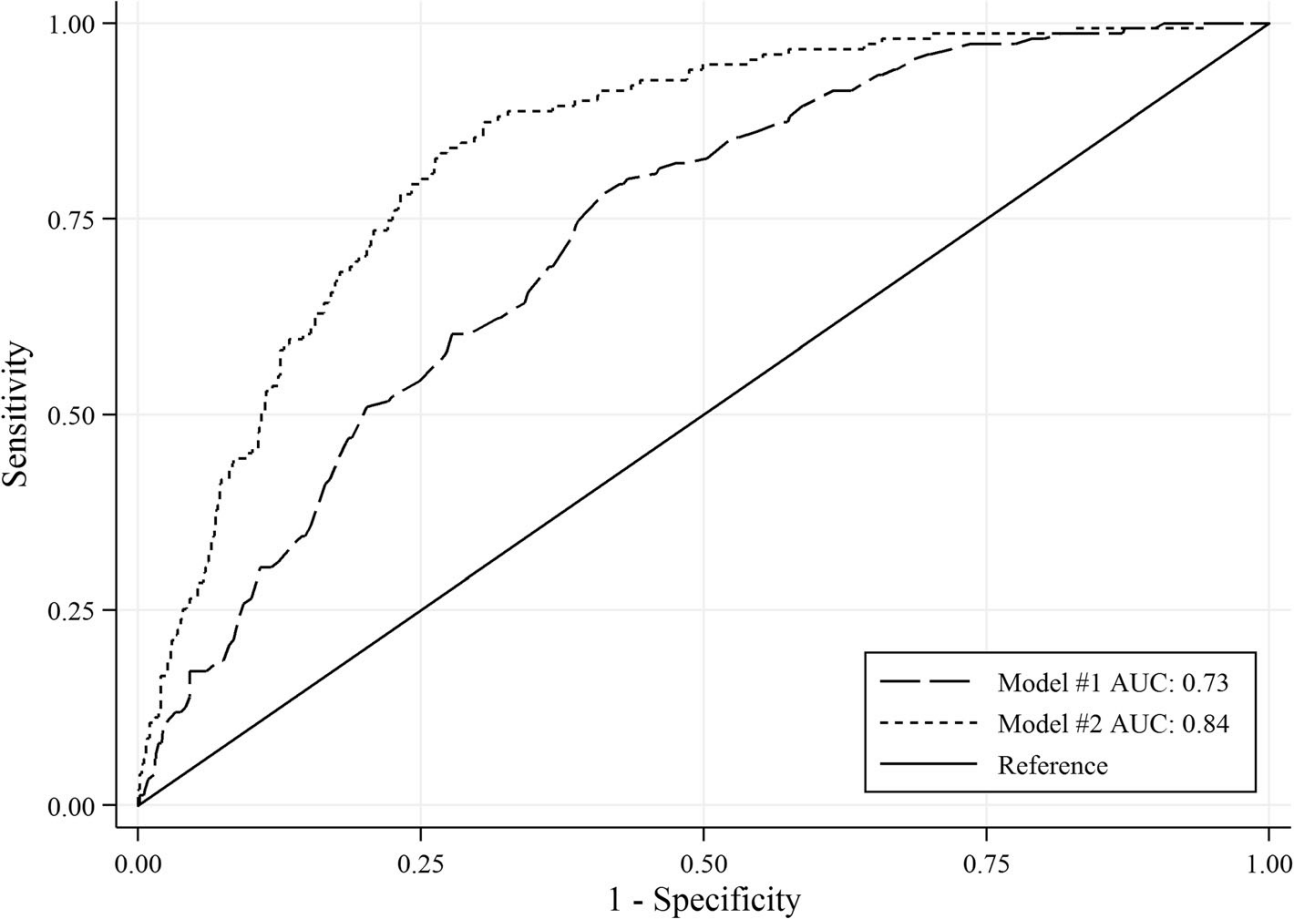
ROC curves for 30-day mortality models based on HDRs only (#1) and HDR plus clinical data (#2). *Note:* The ROC curve is a plot of sensitivity versus 1 − specificity (often called the false-positive rate) that offers a summary of sensitivity and specificity across a range of cut points for a continuous predictor. The area under the curve (AUC) ranges from 0.5 (no discrimination) to a theoretical maximum of 1 (perfect discrimination). *Abbreviations:* ROC, receiver operation characteristic; Model #1 AUC, area under curve for model based on HDR variables; Model #2 AUC, area under curve for model based on HDR plus OPD plus medical charts variables

The probability of 30-day mortality started increasing after the age of 70-80, with no gender differences. Of the HDR variables, only cerebrovascular disease was included in the final model (odds ratio = 1.96, *p* < 0.001). Of the variables retrieved from medical charts, atrial fibrillation and sinus rhythm with a heart rate ≥90 bpm were associated with a higher risk of mortality, as well as low systolic blood pressure at hospital admission. Serum creatinine levels between 0.8 and 1.1 mg/dL were associated with a lower risk of mortality. The inclusion of information from medical charts significantly improved the discrimination of the model (AUC = 0.84 vs. 0.73, *p* < 0.001; LR *χ*^*2*^ = 122.41, *p* < 0.001) and its goodness of fit (pseudo *R*^*2*^ = 0.22 vs. 0.08, AIC = 691.37 vs. 793.56). Figure 3 illustrates ROC curves for models #1 and #2.

#### Thirty-day readmission

**Fig. 4:**
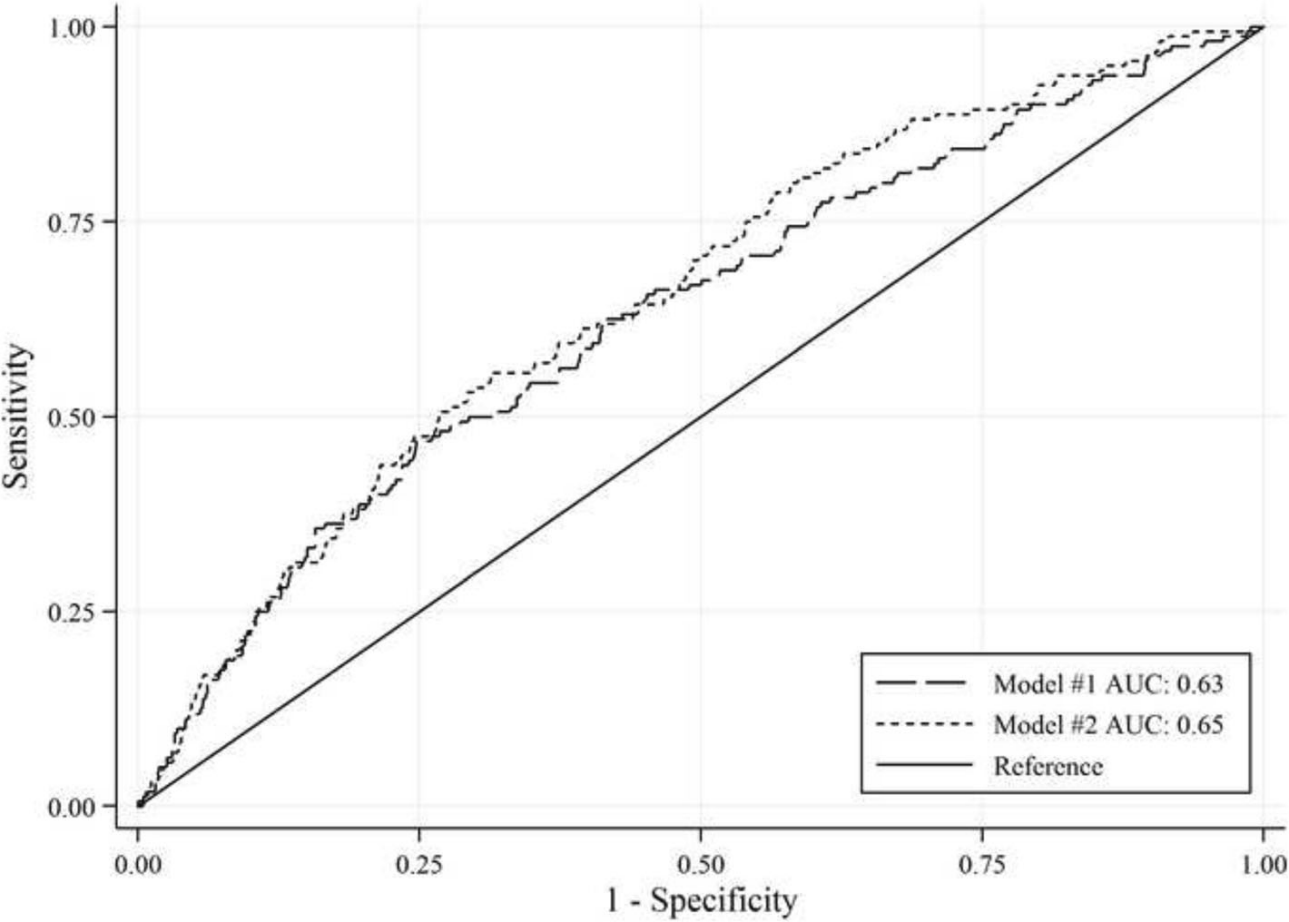
ROC curves for 30-day readmission models based on HDRs only (#1) and HDR plus clinical data (#2)

Older age, longer hospital stay, a history of acute myocardial infarction and low systolic blood pressure levels at hospital admission were associated with a higher risk of 30-day readmission, while gender was unrelated to the outcome. The inclusion of systolic blood pressure did not improve the discrimination (AUC = 0.65 vs. 0.63, *p* = 0.08; LR *χ*^*2*^ = 7.91, *p* = 0.02) and the goodness of fit of the model (pseudo *R*^*2*^ = 0.04 vs. 0.03, AIC = 854.95 vs. 862.86). Figure 4 illustrates ROC curves for models #1 and #2.

None of the data on filled prescriptions were retained in the final risk-adjustment models of 30-day mortality and readmission.

## Discussion

In this large, comprehensive, regional study of patients with HF across three hospitals, our results indicate that adding specific clinical variables retrieved from medical charts significantly contributed to the discrimination of HDR-only models for 30-day mortality, but not for 30-day readmissions. These results confirm the findings of other studies that highlighted the benefits of extracting detailed information from medical records to enhance the discrimination of models for 30-day mortality [8, 10, 12, 14, 15, 24].

Consistent with previous studies [20, 25–34], we found important clinical variables that were significantly associated with 30-day mortality. In particular, the predictors of mortality after hospital admission for HF included: atrial fibrillation, sinus rhythm with a heart rate ≥90 bpm, serum creatinine, and low systolic blood pressure, which also was associated with a higher risk of readmission. Atrial fibrillation is a common morbidity in HF and both conditions are associated with poor outcomes [35] and cardiovascular mortality [25]. Higher heart rate is a marker of poor cardiac function and has been associated with increased risk of mortality as an independent factor [36]. Values of serum creatinine in the range 0.8 and 1.1 mg/dL, which represent good renal function, were protective for 30-day mortality after hospital admission for HF; on the contrary, alteration of serum creatinine level (a marker of renal insufficiency) is often related with HF because of common risk factors and pathogenetic mechanism and is associated with an increasing risk of morbidity and mortality [31, 37]. Lastly, low systolic blood pressure is a marker of poor cardiac output in HF and thus could identify a higher-risk patient [20]. These results demonstrate that it may be useful to collect a relatively small number of specified clinical variables from medical charts that influence 30-day mortality following HF.

The risk-adjustment models for 30-day readmission based on HDRs only and HDRs plus clinical data had a lower discrimination than the mortality models, and adding clinical data did not significantly improve the model, as highlighted by other authors [6, 14]. This result suggests that the data considered for this study, either administrative or clinical, did not adequately predict this outcome. A systematic review found that most of readmission risk prediction models, whether designed for comparative or clinical purposes, perform poorly, and suggested that factors associated with readmission risk may differ according to the setting and population being studied [38]. In our study, given that differences in the case mix of the study patients were not substantial, it is likely that the variables that we collected did not describe sufficiently the patient’s clinical complexity, or that the discrimination of the model for readmissions depended only partially on characteristics regarding patient’s severity and comorbidities. Hospital readmissions may be influenced by other factors, such as quality of care and organizational structure and processes, that were not evaluated in this study [6, 38–40]. As an example, many studies have highlighted the effectiveness of multidisciplinary interventions pre and post discharge on readmissions of patients with HF [2, 41–44]. In order to make readmission models clinically more useful [15, 38], efforts are thus needed to identify variables that may play an important role in predicting this outcome.

An important finding from this study is that previous drug prescriptions had no impact on either 30-day mortality or readmission risk-adjustment models. This may reflect the fact that comorbidities derived from HDRs (either in the index hospitalization or in the previous two years) were comprehensive, and data about drug utilization reflecting the presence of these diseases did not add relevant information to the models. However, a recent study found that inpatient medications, such as insulin, antipsychotics and other drugs with many adverse effects, are associated with a higher risk of 30-day readmission [45]. This information demonstrates that efforts to extract previous drug prescriptions may not be warranted to increase the discrimination of 30-day readmission models for patients with HF, but that data on drugs used during hospital care may substantially enhance their predictive value.

The results of this study should be interpreted in light of its strengths and limitations. This is the first study conducted in Italy that evaluated the contribution of clinical variables and data on drug prescriptions in risk-adjustment models for HF quality of care. Moreover, we investigated a very large number of clinical variables. Limitations include, first, the potential lack of generalizability to other settings; however, this study included all patients from one of the largest Italian LHAs and it is likely that our findings would be generalizable to other regions or countries with a population composition and healthcare delivery system similar to those of this study. Second, data recording and accuracy might have differed to some degree among the three hospitals under study, thus affecting the reliability and validity of results. Third, we did not consider some variables that have been shown to be predictive of mortality and readmission among patients with HF (e.g., body mass index, left ventricular ejection fraction, brain natriuretic peptide, etc.) [15] because they were scarcely reported in medical charts and, when present, mostly measured after medical interventions. Overall, the strengths of this study largely outweigh the limitations.

## Conclusions

In the present study we identified clinical data that significantly improved the discrimination and goodness of fit of a risk-adjustment model based on HDRs to predict 30-day mortality following HF. Because the Italian Ministry of Health has planned to enhance, by 2017, the HDRs Database with the addition of clinical data for specific diseases and procedures, our results identified three “candidate” variables (i.e., systolic blood pressure, heart rhythm, and serum creatinine) that might be easily included in the HDRs to characterize the severity in patients with HF and improve the prediction of 30-day mortality.

Further research is needed to understand the contribution of some additional variables such as organizational and environmental factors, social support, substance abuse and functional status, to predicting 30-day readmissions in patients with HF.

## Additional files

Additional file 1: Description of data sources. (PDF 9 kb) Additional file 2: List of comorbidities retrieved from the Hospital Discharge Records Database. (PDF 16 kb) Additional file 3: Primary data analyzed for this study. (XLS 901 kb)

## Abbreviations

AGENAS: *Agenzia Nazionale per i Servizi Sanitari Regionali*
AIC: Akaike information criterion
AUC: Area under the ROC curve
HDR: Hospital discharge record
HF: Heart failure
ICD: Implantable cardioverter defibrillator
LHA: Local healthcare authority
LR: Likelihood ratio
OPD: Outpatient pharmaceutical database
PNE: *Programma Nazionale Esiti*
ROC: Receiver operation characteristic
