# Supplementary material for: Risk-adjustment models for heart failure patients’ 30-day mortality and readmission rates: the incremental value of clinical data abstracted from medical charts beyond hospital discharge record
Source: BMC Health Serv Res. 2016 Sep 6;16(1):473. doi: 10.1186/s12913-016-1731-9 (PMC5012069; doi:10.1186/s12913-016-1731-9)
Supplement: Additional file 1: — Description of data sources. (PDF 9 kb) [file 12913_2016_1731_MOESM1_ESM.pdf]

Description of data sources

Hospital Discharge Records Database

The Hospital Discharge Records Database includes demographic characteristics, admission referral source, admission and discharge dates, main diagnosis, up to five secondary diagnoses, up to six interventions, and discharge status. Hospital Discharge Records are sent by all public and private hospitals to the Regional Authority, and on a regular basis from the Regional Authority to the Ministry of Health after data quality control. Since 1995 the Diagnosis Related Group system has been systematically used to allocate funds to hospitals and to monitor quality of care and outcomes.

Regional Mortality Register Database

The Regional Mortality Register Database contains information on patients' demographic characteristics as well as date, place and cause of death (classified in the ICD-9-CM).

Regional Health Authority Outpatient Pharmaceutical Database

The Regional Health Authority Outpatient Pharmaceutical Database contains information on patients (identification number, gender and age), prescriptions (substance name, ATC System code—version 2013, trade name, date of prescription filling, and number of packages) and prescribers; it does not contain information on the average daily amount of drug that is actually prescribed to the patient. This register includes drugs reimbursed by the healthcare system that are prescribed by the primary care physician or a specialist, or directly delivered by the hospital pharmacies.

Medical charts

In Emilia-Romagna all hospital medical charts, although still paper-based, are scanned in "read-only" format.
