# Supplementary material for: Risk-adjustment models for heart failure patients’ 30-day mortality and readmission rates: the incremental value of clinical data abstracted from medical charts beyond hospital discharge record
Source: BMC Health Serv Res. 2016 Sep 6;16(1):473. doi: 10.1186/s12913-016-1731-9 (PMC5012069; doi:10.1186/s12913-016-1731-9)
Supplement: Additional file 2: — List of comorbidities retrieved from the Hospital Discharge Records Database. (PDF 16 kb) [file 12913_2016_1731_MOESM2_ESM.pdf]

List of comorbidities retrieved from the Hospital Discharge Records Database

Condition	ICD-9-CM codes	
	Heart failure episode	Hospital admissions in the previous 2 years
Malignant tumors	140.0–208.9, V10	140.0–208.9, V10
Diabetes*		250.0–250.9
Disorders of lipoid metabolism*		272
Obesity	278.0	278.0
Hematologic diseases	280–285, 288, 289	280–285, 288, 289
Hypertensive diseases*		401–405
Previous AMI	412	410, 412
Other forms of ischemic heart disease		411, 413, 414
Ill-defined descriptions and complications of heart disease		429
Rheumatic heart disease	393–398	391, 393–398
Cardiomyopathies	425	425
Other cardiac diseases	745, V15.1, V42.2, V43.2, V43.3, V45.0	745, V15.1, V42.2, V43.2, V43.3, V45.0
Conduction disorders and cardiac dysrhythmias		426, 427
Cerebrovascular diseases	433, 437, 438	430–432, 433, 434, 436, 437, 438
Vascular diseases	440–448 (except 441.1, 441.3, 441.5, 441.6, 444), 557.1	440–448, 557
COPD*		491–492, 494, 496
Chronic nephropathies	582–583, 585–588	582–583, 585–588
Chronic diseases of liver, pancreas and intestine	571–572, 577.1–577.9, 555, 556	571–572, 577.1–577.9, 555, 556
Previous bypass	V45.81	V45.81, 36.1
Previous PCI	V45.82	V45.82, 36.0, 00.66
Other surgery of the heart		35, 37.0, 37.1, 37.3, 37.4, 37.5, 37.6, 37.9
Other surgery of great vessels		38–39.5 (except 38.01, 38.02, 38.5, 38.11, 38.12, 38.31, 38.32, 38.93)
Previous diagnosis of heart failure		428

*These conditions were not considered at the index episode, because they are not likely to be reported for very severe patients.
Abbreviations: AMI, acute myocardial infarction; COPD, chronic obstructive airway disease; PCI, percutaneous coronary intervention.
